# Supplementary material for: Comparing the clinical and economic efficiency of four natural surfactants in treating infants with respiratory distress syndrome
Source: PLoS One. 2023 Jun 30;18(6):e0286997. doi: 10.1371/journal.pone.0286997 (PMC10313081; doi:10.1371/journal.pone.0286997)
Supplement: S1 File — (DOCX) [file pone.0286997.s001.docx]

**S1: How to calculate MABAC and CRITIC methods in neonates <=32**

**S1-1: CRITIC method calculations**

**Table S1.1 Decision matrix**

|  | re-dosing rate | average length of stay | average direct treatment cost | medical referral rate | live discharge rate | DALY per 1,000 infants | number of infants in need of mechanical ventilation |
| --- | --- | --- | --- | --- | --- | --- | --- |
|  | I1 | I2 | I3 | I4 | I5 | I6 | I7 |
| Alveofact | 1.75 | 13.91 | 675.7071394 | 20 | 73 | 14238.22632 | 116 |
| BLES | 1.4 | 17.27 | 266.2549105 | 136 | 580 | 12289.52557 | 610 |
| Curosurf | 1.35 | 21.28 | 402.1170118 | 275 | 2404 | 11689.06809 | 2313 |
| Survanta | 1.35 | 17.35 | 335.4233364 | 117 | 794 | 11120.09871 | 754 |

**Table S1.2 Normalized Decision matrix**

| R | I1 | I2 | I3 | I4 | I5 | I6 | I7 |
| --- | --- | --- | --- | --- | --- | --- | --- |
| Alveofact | 1 | 0 | 1 | 0 | 0 | 1 | 0 |
| BLES | 0.125 | 0.455902307 | 0 | 0.454901961 | 0.217503218 | 0.375041371 | 0.224852071 |
| Curosurf | 0 | 1 | 0.331814292 | 1 | 1 | 0.182471485 | 1 |
| Survanta | 0 | 0.466757123 | 0.168929172 | 0.380392157 | 0.309309309 | 0 | 0.290395995 |
|  |  |  |  |  |  |  |  |

**Table S1.3 Values of CRITIC parameters**

| Standard deviation (σ) | 0.4827 | 0.4088 | 0.4380 | 0.4121 | 0.4321 | 0.4349 | 0.4323 |
| --- | --- | --- | --- | --- | --- | --- | --- |
| Value of C | 1.3879 | 0.8493 | 1.0947 | 0.8921 | 1.0687 | 1.0623 | 1.0743 |
| **Weight** | **0.1868** | **0.1143** | **0.1473** | **0.1200** | **0.1438** | **0.1429** | **0.1446** |

**S1-2: MABAC method calculations**

**Table S1.4 Normalized Decision matrix (MABAC)**

|  | re-dosing rate | average length of stay | average direct treatment cost | medical referral rate | live discharge rate | DALY per 1,000 infants | number of infants in need of mechanical ventilation |
| --- | --- | --- | --- | --- | --- | --- | --- |
|  | 0 | 1 | 0 | 1 | 0 | 0 | 1 |
| Alveofact | 0.875 | 0.5440 | 1 | 0.5450 | 0.2175 | 0.6249 | 0.7751 |
| BLES | 1 | 0 | 0.6681 | 0 | 1 | 0.8175 | 0 |
| Curosurf | 1 | 0.5332 | 0.8310 | 0.6196 | 0.3093 | 1 | 0.7096 |
| Survanta | 0 | 1 | 0 | 1 | 0 | 0 | 1 |

**Table S1.5 weighted normalized matrix (V)**

| V | I1 | I2 | I3 | I4 | I5 | I6 | I7 |
| --- | --- | --- | --- | --- | --- | --- | --- |
| Alveofact | 0.1868 | 0.2286 | 0.1473 | 0.2402 | 0.1438 | 0.143 | 0.2892 |
| BLES | 0.3503 | 0.1765 | 0.2947 | 0.1855 | 0.1751 | 0.2323 | 0.2567 |
| Curosurf | 0.3736 | 0.1143 | 0.2458 | 0.1201 | 0.2877 | 0.2599 | 0.1446 |
| Survanta | 0.3736 | 0.1753 | 0.2698 | 0.1945 | 0.1883 | 0.286 | 0.2472 |

**Table S1.6 Distance of alternatives from (BAA) matrix**

| Q | I1 | I2 | I3 | I4 | I5 | I6 | I7 |
| --- | --- | --- | --- | --- | --- | --- | --- |
| Alveofact | -0.122 | 0.06 | -0.0843 | 0.0606 | -0.0484 | -0.08 | 0.0622 |
| BLES | 0.0411 | 0.0079 | 0.063 | 0.0059 | -0.0171 | 0.0094 | 0.0297 |
| Curosurf | 0.0645 | -0.0543 | 0.0141 | -0.06 | 0.0955 | 0.037 | -0.082 |
| Survanta | 0.0645 | 0.0066 | 0.0382 | 0.0149 | -0.0039 | 0.0631 | 0.0202 |

**Table S1.7 The values of** $\boldsymbol{S}_{\boldsymbol{i}}$

| Type of Surfactant | $\boldsymbol{S}_{\boldsymbol{i}}$ (=<32) | Rank |
| --- | --- | --- |
| Alveofact | -0.152136722 | 4 |
| BLES | 0.140056915 | 2 |
| Curosurf | 0.01485928 | 3 |
| Survanta | 0.203576162 | 1 |
