# Supplementary material for: Comparing the clinical and economic efficiency of four natural surfactants in treating infants with respiratory distress syndrome
Source: PLoS One. 2023 Jun 30;18(6):e0286997. doi: 10.1371/journal.pone.0286997 (PMC10313081; doi:10.1371/journal.pone.0286997)
Supplement: S2 File — (DOCX) [file pone.0286997.s002.docx]

**S2: How to calculate MABAC and CRITIC methods in neonates >32**

**S2-1: CRITIC method calculations**

**Table S2.1 Decision matrix**

|  | re-dosing rate | average length of stay | average direct treatment cost | medical referral rate | live discharge rate | DALY per 1,000 infants | number of infants in need of mechanical ventilation |
| --- | --- | --- | --- | --- | --- | --- | --- |
|  | I1 | I2 | I3 | I4 | I5 | I6 | I7 |
| Alveofact | 1.5 | 9.89 | 580.1830151 | 12 | 103 | 3671.546699 | 47 |
| BLES | 1.31 | 9.3 | 250.4575547 | 139 | 692 | 2637.82126 | 228 |
| Curosurf | 1.24 | 10.44 | 369.1220951 | 172 | 2854 | 3294.591616 | 1237 |
| Survanta | 1.24 | 10.24 | 308.6833598 | 111 | 1249 | 2819.718295 | 557 |

**Table S2.2 Normalized Decision matrix**

| R | I1 | I2 | I3 | I4 | I5 | I6 | I7 |
| --- | --- | --- | --- | --- | --- | --- | --- |
| Alveofact | 1 | 0.51754386 | 1 | 0 | 0 | 1 | 0 |
| BLES | 0.269230769 | 0 | 0 | 0.79375 | 0.214103962 | 0 | 0.15210084 |
| Curosurf | 0 | 1 | 0.359888922 | 1 | 1 | 0.635343131 | 1 |
| Survanta | 0 | 0.824561404 | 0.176588745 | 0.61875 | 0.416575791 | 0.175962618 | 0.428571429 |

**Table S2.3 Values of CRITIC parameters**

| Standard deviation (σ) | 0.4724 | 0.4383 | 0.4360 | 0.4312 | 0.4299 | 0.4525 | 0.4405 |
| --- | --- | --- | --- | --- | --- | --- | --- |
| Value of C | 1.2902 | 0.7266 | 1.0743 | 0.6845 | 1.0187 | 0.9905 | 1.0657 |
| **Weight** | **0.1883** | **0.1060** | **0.1568** | **0.0999** | **0.1487** | **0.1445** | **0.1555** |

**S2-2: MABAC method calculations**

**Table S2.4 Normalized Decision matrix (MABAC)**

|  | re-dosing rate | average length of stay | average direct treatment cost | medical referral rate | live discharge rate | DALY per 1,000 infants | number of infants in need of mechanical ventilation |
| --- | --- | --- | --- | --- | --- | --- | --- |
|  | 0 | 0.4825 | 0 | 1 | 0 | 0 | 1 |
| Alveofact | 0.7308 | 1 | 1 | 0.2063 | 0.2141 | 1 | 0.8479 |
| BLES | 1 | 0 | 0.6401 | 0 | 1 | 0.3647 | 0 |
| Curosurf | 1 | 0.1754 | 0.8234 | 0.3813 | 0.4166 | 0.824 | 0.5714 |
| Survanta | 0 | 0.4825 | 0 | 1 | 0 | 0 | 1 |

**Table S2.5 weighted normalized matrix (V)**

| V | I1 | I2 | I3 | I4 | I5 | I6 | I7 |
| --- | --- | --- | --- | --- | --- | --- | --- |
| Alveofact | 0.1883 | 0.1573 | 0.1568 | 0.1998 | 0.1487 | 0.1446 | 0.3111 |
| BLES | 0.326 | 0.2121 | 0.3136 | 0.1205 | 0.1805 | 0.2892 | 0.2875 |
| Curosurf | 0.3767 | 0.1061 | 0.2572 | 0.0999 | 0.2974 | 0.1973 | 0.1556 |
| Survanta | 0.3767 | 0.1247 | 0.2859 | 0.138 | 0.2106 | 0.2637 | 0.2445 |

**Table S2.6 Distance of alternatives from (BAA) matrix**

| Q | I1 | I2 | I3 | I4 | I5 | I6 | I7 |
| --- | --- | --- | --- | --- | --- | --- | --- |
| Alveofact | -0.1172 | 0.0123 | -0.0884 | 0.0648 | -0.054 | -0.071 | 0.0696 |
| BLES | 0.0205 | 0.0672 | 0.0684 | -0.0145 | -0.022 | 0.0732 | 0.046 |
| Curosurf | 0.0712 | -0.039 | 0.012 | -0.0351 | 0.0949 | -0.019 | -0.0859 |
| Survanta | 0.0712 | -0.02 | 0.0407 | 0.003 | 0.0081 | 0.0478 | 0.003 |

**Table S2.7 The values of** $\boldsymbol{S}_{\boldsymbol{i}}$

| Type of Surfactant | $\boldsymbol{S}_{\boldsymbol{i}}$ (>32) | Rank |
| --- | --- | --- |
| Alveofact | -0.183981929 | 4 |
| BLES | 0.238804661 | 1 |
| Curosurf | -0.000511128 | 3 |
| Survanta | 0.153497584 | 2 |
