# Supplementary material for: Comparing the clinical and economic efficiency of four natural surfactants in treating infants with respiratory distress syndrome
Source: PLoS One. 2023 Jun 30;18(6):e0286997. doi: 10.1371/journal.pone.0286997 (PMC10313081; doi:10.1371/journal.pone.0286997)
Supplement: S3 File — (DOCX) [file pone.0286997.s003.docx]

**S3:**

| **Table S3.1. Baseline characteristics, abnormalities, and risk factors** | | | | | |
| --- | --- | --- | --- | --- | --- |
| **Demographic characteristics and risk factors of neonates** | | | | | |
| ***P-Value*** | ***Survanta*** | ***Curosurf*** | ***BLES*** | ***Alveofact*** | ***Variables*** |
|  | ***(N =2949)*** | ***(N =7788)*** | ***(N =2124)*** | ***(N =308)*** |  |
|  | | | | | **Gender** |
| 0.11 | 1359(46.08) | 3167(40.66) | 876(41.24) | 131(42.53) | Female |
|  | 1589(53.88) | 4613(59.23) | 1245(58.61) | 175(56.81) | Male |
|  | 1(0.03) | 8(0.10) | 3(0.14) | 2(0.64) | Ambiguous genitalia |
|  | | | | | **Gestational age** |
| 0.07 | 32.44(±4.44) | 32.27 (±4.58) | 32.19 (±4.77) | 32.1 (±4.41) | Mean, wk |
|  | 1448(49.10) | 4389(56.35) | 1213(57.10) | 177(57.46) | ≤ 32 weeks |
|  | 1501(50.89) | 3399(43.64) | 911(42.89) | 131(42.53) | > 32 weeks |
|  | | | | | **Birth weight** |
| 0.09 | 1908.91(±849) | 1906.37(±839) | 1836.06(±852) | 1856.42(±715) | Mean, gr |
|  | 1465(49.67) | 3275(42.05) | 976(45.95) | 124(40.25) | ≤ 1500gr |
|  | 1484(50.32) | 4513(57.94) | 1146(53.95) | 184(59.74) | > 1500gr |
|  | | | | | **Delivery method** |
| 0.16 | 2290(77.65) | 5752(73.85) | 1567(73.77) | 233(75.64) | Cesarean |
|  | 652(22.10) | 1991(25.56) | 547(25.75) | 73(23.70) | Vaginal |
|  | 7(0.23) | 43(0.55) | 10(0.47) | 2(0.64) | VBAC |
|  | | | | | **Apgar score** |
| 0.18 | 6.95 | 6.93 | 6.94 | 6.94 | First minute Apgar |
| 0.13 | 8.32 | 8.33 | 8.33 | 8.28 | Five-minute Apgar |
|  | | | | | **Number of newborns in one birth** |
| 0.29 | 2561(86.84) | 6696(85.97) | 1839(86.58) | 254(82.46) | Singleton birth |
|  | 388(13.15) | 951(12.21) | 259(12.19) | 38(12.33) | Multiple births |
|  | 338(11.46) | 839(10.77) | 222(10.45) | 33(10.71) | Twins |
|  | 50(1.69) | 112(1.43) | 37(1.74) | 5(1.62) | More than twins |
|  | 45(1.52) | 141(1.81) | 26(1.22) | 16(5.19) | Missing data |
|  | | | | | **Other risk factors** |
| 0.75 | 144(4.88) | 388(4.98) | 85(4.00) | 14(4.54) | PROM (premature rupture of membrane) |
| 0.80 | 25(0.84) | 72(0.92) | 23(1.08) | 2(0.64) | Placental abruption |
| 0.72 | 5(0.16) | 36(0.46) | 6(0.28) | 1(0.32) | Placenta Accreta |
| 0.34 | 147(4.98) | 509(6.53) | 147(6.92) | 17(5.51) | MSAF (meconium-stained amniotic fluid) |
| 0.28 | 189(6.40) | 406(5.21) | 134(6.30) | 13(4.22) | Periventricular leukomalacia (PVL) |
| 0.19 | 214(7.25) | 568(7.29) | 147(6.92) | 22(7.21) | Intraventricular hemorrhage (IVH) |
| 0.55 | 208(7.05) | 512(6.57) | 162(7.62) | 21(6.81) | Patent ductus arteriosus (PDA) |
| 0.69 | 223(7.56) | 627(8.05) | 164(7.72) | 23(7.46) | Pneumothorax |
| 0.31 | 72(2.44) | 346(4.44) | 81(3.81) | 9(2.92) | Pulmonary hemorrhage |
| **Mother's underlying diseases, and risk factors related to the mother** | | | | | |
| ***P-Value*** | ***Survanta*** | ***Curosurf*** | ***BLES*** | ***Alveofact*** | ***Variables*** |
| 0.51 | 419(14.20) | 1103(14.16) | 262(12.33) | 43(13.96) | Gestational diabetes |
| 0.43 | 109(3.69) | 292(3.74) | 74(3.48) | 4(1.29) | Chronic hypertension |
| 0.54 | 250(8.47) | 681(8.74) | 214(10.07) | 20(6.49) | Pre-eclampsia/eclampsia |
| 0.37 | 22(0.74) | 54(0.69) | 6(0.28) | 1(0.32) | Heart disease |
| 0.86 | 3(0.10) | 10(0.12) | 2(0.09) | 0(0) | Hepatitis B |
| 0.72 | 23(0.77) | 54(0.69) | 12(0.56) | 0(0) | History of stillbirth |
| 0.82 | 4(0.13) | 3(0.03) | 1(0.04) | 0(0) | Mother's smoking in the last pregnancy |
| 0.29 | 2138)72.49( | 5112)65.63( | 1428(67.23( | 221(71.75( | Steroid injection |
|  | **Congenital abnormality** | | | | |
| ***P-Value*** | ***Survanta*** | ***Curosurf*** | ***BLES*** | ***Alveofact*** | ***The type of abnormality*** |
| - | 70(2.37) | 210(2.69) | 36(1.69) | 6(1.94) | Congenital heart defect |
| - | 2(0.06) | 5(0.06) | 3(0.14) | 0(0) | Skin abnormalities |
| - | 23(0.77) | 31(0.39) | 6(0.28) | 1(0.32) | Eye/ear/nose/head/neck abnormalities |
| - | 13(0.44) | 38(0.48) | 7(0.32) | 0(0) | Musculoskeletal abnormalities |
| - | 18(0.61) | 45(0.57) | 7(0.32) | 3(0.97) | Urinary-/genital abnormalities |
| - | 7(0.23) | 46(0.59) | 11(0.51) | 2(0.64) | Gastrointestinal abnormalities |
| - | 17(0.57) | 48(0.61) | 13(0.61) | 3(0.97) | Respiratory abnormalities |
| - | 10(0.33) | 38(0.48) | 7(0.32) | 1(0.32) | Neurological abnormalities |
| - | 4(0.13) | 8(0.10) | 2(0.09) | 0(0) | Chromosomal abnormalities |
| - | 29(0.98) | 48(0.61) | 16(0.75) | 4(1.29) | Other abnormalities |
| 0.26 | 193(6.54) | 517(6.63) | 108(5.08) | 20(6.49) | The total number of abnormalities |
| **Therapeutic interventions** | | | | | |
| ***P-Value*** | ***Survanta*** | ***Curosurf*** | ***BLES*** | ***Alveofact*** | ***The type of abnormality*** |
| 0.84 | 1643 (55.71) | 4092 (52.54) | 1058 (49.81) | 143 (46.42) | Use of non-invasive ventilation/ NCPAP |
|  | | | | | **Method of administration (first dose)** |
| 0.21 | 1397(47.37) | 3412(43.81) | 945(44.91) | 132 (42.85) | INSURE |
|  | 46(0.15) | 69(0.88) | 16(0.75) | 2(0.64) | MIST (surfactant prescription without intubation) |
|  | 716(24.27) | 1996(25.62) | 496(23.35) | 71(23.05) | During mechanical ventilation |
|  | 788(26.72) | 2309(29.64) | 667(31.40) | 103(33.44) | Missing data |
