# Supplementary material for: Comparing the clinical and economic efficiency of four natural surfactants in treating infants with respiratory distress syndrome
Source: PLoS One. 2023 Jun 30;18(6):e0286997. doi: 10.1371/journal.pone.0286997 (PMC10313081; doi:10.1371/journal.pone.0286997)
Supplement: S4 File — (DOC) [file pone.0286997.s004.doc]

STROBE Statement—Checklist of items that should be included in reports of ***cross-sectional studies***

| **Section/topic** | Item No | Recommendation | Page/Line | Comments:  “Relevant text in the manuscript” |
| --- | --- | --- | --- | --- |
| **Title and abstract** | 1 | (*a*) Indicate the study’s design with a commonly used term in the title or the abstract | P2/L17 | Study design is indicated in the Methods section of the abstract:  *“The research was a cross-sectional, retrospective study…”* |
|  |  | (*b*) Provide in the abstract an informative and balanced summary of what was done and what was found | P2/L16  P2/L19  P2/L21-23  P2/L27-33 | This information is stated in the study abstract (study objective described, method and results described):  what was done (objective and method):   - *“To compare four common surfactants in the health market of Iran to determine the best surfactant according to the selected criteria”* - *“To rank the surfactants used, the following indicators were measured…”* - *“The CRITIC (criteria importance through intercriteria correlation) method was used to determine the weight of the indicators, and MABAC (multi-attributive border approximation area comparison) was used to prioritize the surfactants.”*   what was found (results):  *“Alveofact was identified as the worst surfactant in infants with either more or less than 32 weeks’ gestation.* *BLES was the best alternative for infants more than 32 weeks’ gestation, whereas* *Survanta was identified as best option for infants with less than 32 weeks’ gestation.”* |
| Introduction | | |  |  |
| Background/rationale | 2 | Explain the scientific background and rationale for the investigation being reported | P3/L54  P4/L76-84 | Rationale and existing literature are stated in the introduction section:  Existing literature*: “Numerous studies have compared the types of surfactants…”*  Rationale: “*According to the unpublished statistics of the Ministry of Health and interviews with policy makers in the field of neonatal health, the prescription of surfactant in neonates has been growing rapidly in Iran in recent years, and on the other hand, surfactant is one of the vital and expensive drugs in Iran's pharmaceutical pharmacopoeia. Therefore, it is important to determine the type of superior surfactant that provides positive clinical results and is also economically viable, as it can help specialists and decision makers to make appropriate decisions. In addition, although many studies have been conducted on various aspects of respiratory distress in neonates and the effect of different surfactants on it, most of the studies in this regard have only described and compared the results by different types of surfactants.”* |
| Objectives | 3 | State specific objectives, including any prespecified hypotheses | P4/L84-87 | A statement at the end of the introduction specifies the specific goals and objectives:  *"This study was conducted with the aim of determining the best and worst surfactants in the treatment of respiratory distress syndrome in infants in Iran's health system. In this research, based on economic and clinical indicators, surfactants have been ranked for the first time with a new method."* |
| Methods | | |  |  |
| Study design | 4 | Present key elements of study design early in the paper | P4/L90-91 | Study design is stated in the first subsection of Methods. Key elements are all described in the methods:  *“This study was a cross-sectional, retrospective research that tried to evaluate the effectiveness of four types of surfactants and rank them.”* |
| Setting | 5 | Describe the setting, locations, and relevant dates, including periods of recruitment, exposure, follow-up, and data collection | P4/91-92 | Settings, contexts, dates of inclusion, are fully described in the Method section, sub-section "2.1 Research design and patient population":  *“The population under study included all infants with RDS who underwent surfactant therapy in Iran in 2018.”* |
| Participants | 6 | (*a*) Give the eligibility criteria, and the sources and methods of selection of participants | P5/L96-104 | The study population and eligibility criteria are described in the method section (sub-section “Research design and patient population"):  *"The data of these infants were extracted using the census method, in fact, sampling was not done, and all eligible infants were included in the study using the census method. The entry and exit criteria were as follows:*  *-Inclusion criteria: All infants with RDS in Iran who had undergone surfactant therapy;*  *-Exclusion criteria: The following cases were excluded from the study: Infants with RDS who were discharged with the consent of their parents (or legal guardians) before full recovery, those who were not primarily diagnosed with RDS, and those with an unidentified gestational age. Also, infants who were referred to another center before surfactant administration were excluded from the study."* |
| Variables | 7 | Clearly define all outcomes, exposures, predictors, potential confounders, and effect modifiers. Give diagnostic criteria, if applicable | P5-8 /L120-186  P5/L112-17 | All the variables considered for statistical analyzes and multi-criteria decision-making are clearly defined in the method section, in the sub-section "2.2 Study variables and measured outcomes":  *" The indicators investigated in this research were extracted from the literature on this topic:*  *1.Redosing rate: A surfactant would be most efficacious when it required less redosing;*  *.*  *.*  *.*  *.*  *7. Number of newborns in need of mechanical ventilation: A large number of newborns in need of mechanical ventilation or a long period of mechanical ventilation could reflect the low efficiency of an injected surfactant.”*  **********  The descriptive variables of the study are reported as follows:  *“To ensure that there is no significant difference in the baseline in the neonates of the four surfactant groups, four indicators of gestational age, birth weight, Apgar 1st minute and Apgar 5th minute were used, which indicate the general health status of the newborns at birth and before surfactant administration; And it was determined in advance that before the administration of surfactant, there was no statistically significant difference in the health status of infants in different surfactant groups.”* |
| Data sources/ measurement | 8* | For each variable of interest, give sources of data and details of methods of assessment (measurement). Describe comparability of assessment methods if there is more than one group | P5/L105-106 | Data collection and measurement was the same for all variables, and is described in the methods section. All data were extracted from a national system in Iran (Iranian Maternal and Neonatal Network (IMAN net)). And the measurement of the variables was based on the values recorded in this network. Relevant explanations are provided in the sub-section "2.1 Research design and patient population":  *“The research data were extracted from the Iranian Maternal and Neonatal Network (IMAN net), as per the necessary permits and with the support of the Iranian Ministry of Health.”* |
| Bias | 9 | Describe any efforts to address potential sources of bias | P5/100-104  P5/L112-117 | We tried to avoid biasing the results as much as possible by applying strict exclusion criteria:  *"-Exclusion criteria: The following cases were excluded from the study: Infants with RDS who were discharged with the consent of their parents (or legal guardians) before full recovery, those who were not primarily diagnosed with RDS, and those with an unidentified gestational age. Also, infants who were referred to another center before surfactant administration were excluded from the study."*  **********  Also, by examining the baseline variables, we made sure that the four investigated groups did not differ significantly in terms of health status before surfactant administration:  *“To ensure that there is no significant difference in the baseline in the neonates of the four surfactant groups, four indicators of gestational age, birth weight, Apgar 1st minute and Apgar 5th minute were used, which indicate the general health status of the newborns at birth and before surfactant administration; And it was determined in advance that before the administration of surfactant, there was no statistically significant difference in the health status of infants in different surfactant groups.”* |
| Study size | 10 | Explain how the study size was arrived at | P5/L96-97 | In this study, the census method was used for the entire eligible population in Iran:  *“…in fact, sampling was not done, and all eligible infants were included in the study using the census method.”* |
| Quantitative variables | 11 | Explain how quantitative variables were handled in the analyses. If applicable, describe which groupings were chosen and why | P5-8 /L120-186 | All variables are explicitly defined in the sub-section "2.2 Study variables and measured outcomes ":  *" The indicators investigated in this research were extracted from the literature on this topic:*  *1.Redosing rate: A surfactant would be most efficacious when it required less redosing;*  *.*  *.*  *.*  *.*  *7. Number of newborns in need of mechanical ventilation: A large number of newborns in need of mechanical ventilation or a long period of mechanical ventilation could reflect the low efficiency of an injected surfactant.”*  And how to use these variables in the analysis, two stages of weighting the variables and ranking them are explained in detail in the sub-section "2.3.1 CRITIC method" and the sub-section "2.3.2 MABAC method". |
| Statistical methods | 12 | (*a*) Describe all statistical methods, including those used to control for confounding | P8-10/ L187-237 | In a very detailed and step-by-step manner, all analysis methods are presented in section "2.3 Data analysis" and its two sub-sections (2.3.1 CRITIC method and 2.3.2 MABAC method). |
|  |  | (*b*) Describe any methods used to examine subgroups and interactions | P5/L108-111 | Analyzes of the target population were performed separately for two subgroups according to gestational age; This is stated in the sub-section "2.1 Research design and patient population":  *“Because the outcomes of surfactant injection could be different among different age groups [21], the data were analyzed separately for two groups of infants: those with a gestational age more than 32 weeks and those with a gestational age less than 32 weeks.”* |
|  |  | (*c*) Explain how missing data were addressed | P5/ L106-108 | The design of the data source used in this research is such that it is mandatory to record information related to many variables:  *“It should be noted that the design of this national network is such that it is mandatory to record information for many variables, so the missing data in this study is minimal.”* |
|  |  | (*d*) If applicable, describe analytical methods taking account of sampling strategy | - | Non applicable |
|  |  | (*e*) Describe any sensitivity analyses | P15/L294-308 | It is explained in detail in sub-section "3.4 Validation of results and sensitivity analysis".  *“In order to examine the obtained results using MABAC, the all type of surfactants will be ranked using two other methods: MAIRCA method, and VIKOR method. And…”* |
| Results | | |  |  |
| Participants | 13* | (a) Report numbers of individuals at each stage of study—eg numbers potentially eligible, examined for eligibility, confirmed eligible, included in the study, completing follow-up, and analysed | P11/L241-245 | This is described at the beginning of result section:  *“According to the inclusion and exclusion criteria of the study, the number of infants studied in this research decreased from 16551 to 13169 cases. ...”* |
|  |  | (b) Give reasons for non-participation at each stage | P11/L242-245 | Based on this item from the checklist, the following text was added to the first sub-section of the results:  *“The number of 1133 babies due to not having RDS problem, 1093 babies due to voluntary discharge, 1100 babies due to uncertain gestational age, and finally 56 cases due to referral to another* *hospital before surfactant administration, all these babies (3382 cases) were excluded from the study.”* |
|  |  | (c) Consider use of a flow diagram | - | Use of a flow diagram was not deemed necessary. |
| Descriptive data | 14* | (a) Give characteristics of study participants (eg demographic, clinical, social) and information on exposures and potential confounders | P11/ L240-251 | All this information is presented in the first sub-section of the results under the title "3.1 Patient demographics and clinical characteristics" in text form and in Table 1. |
|  |  | (b) Indicate number of participants with missing data for each variable of interest | - | Non applicable (due to mandatory data registration for variables in the data source used in the research) |
| Outcome data | 15* | Report numbers of outcome events or summary measures | P11/L251  P12/L260 | All numbers are reported in Table1 and Tables2. |
| Main results | 16 | (*a*) Give unadjusted estimates and, if applicable, confounder-adjusted estimates and their precision (eg, 95% confidence interval). Make clear which confounders were adjusted for and why they were included | P11/L251 | Baseline variables that were necessary to determine the health status of infants before surfactant administration are presented in Table 1, and P-value values are written in the last row of the table. |
|  |  | (*b*) Report category boundaries when continuous variables were categorized | - | Non applicable |
|  |  | (*c*) If relevant, consider translating estimates of relative risk into absolute risk for a meaningful time period | - | Non applicable |
| Other analyses | 17 | Report other analyses done—eg analyses of subgroups and interactions, and sensitivity analyses | P13-15/ L261-308 | All the analyzes are presented in Tables 3-7, separately for two age groups of babies less than and more than 32 weeks. |
| Discussion | | |  |  |
| Key results | 18 | Summarise key results with reference to study objectives | P16-21/ L310-414 | In the first paragraph of the discussion section, the main purpose of the research is stated and the discussion section is presented in two general parts based on it. |
| Limitations | 19 | Discuss limitations of the study, taking into account sources of potential bias or imprecision. Discuss both direction and magnitude of any potential bias | P21-22/ L465-486 | The limitations of the research are written in detail in the last paragraph of the discussion section:  *“As with all retrospective studies, one of the inherent limitations of this study was the heterogeneity of the patients in the groups under investigation.”* |
| Interpretation | 20 | Give a cautious overall interpretation of results considering objectives, limitations, multiplicity of analyses, results from similar studies, and other relevant evidence | P16-21/ L310-414 | Based on the purpose of the research, the results were expressed cautiously in two parts (general comparison of surfactants, and comparison of surfactants based on selected indicators), and were compared with similar and opposite studies. |
| Generalisability | 21 | Discuss the generalisability (external validity) of the study results | P16-21/ L310-414 | Similar results from other studies from Iran with a smaller sample size were discussed. Also, several studies with similar results from other countries were used in the discussion (for example, the study of Fujii et al. from the United States, the study of Bloom et al. from Columbia, Akar et al. from Turkey, etc.) |
| Other information | | |  |  |
| Funding | 22 | Give the source of funding and the role of the funders for the present study and, if applicable, for the original study on which the present article is based | - | Funding information were displayed upon submission but not included in the manuscript, as requested. |

*Give information separately for exposed and unexposed groups.

**Note:** An Explanation and Elaboration article discusses each checklist item and gives methodological background and published examples of transparent reporting. The STROBE checklist is best used in conjunction with this article (freely available on the Web sites of PLoS Medicine at http://www.plosmedicine.org/, Annals of Internal Medicine at http://www.annals.org/, and Epidemiology at http://www.epidem.com/). Information on the STROBE Initiative is available at www.strobe-statement.org.
