# Supplementary material for: Comparing the clinical and economic efficiency of four natural surfactants in treating infants with respiratory distress syndrome
Source: PLoS One. 2023 Jun 30;18(6):e0286997. doi: 10.1371/journal.pone.0286997 (PMC10313081; doi:10.1371/journal.pone.0286997)
Supplement: Attachment — Submitted filename: comments.docx [file pone.0286997.s006.docx]

I appreciate authors who have brought into the light about unattended facet of newborn care.

There should be a STROBE checklist attached with (manuscript) to address all the aspects of observational studies.

Abstract

1. CRITIC/MABAC/BLES acronym should be expanded
2. The standard terminology for Live discharge rate should be survival at discharge.
3. Some scores denoting the inferiority of Alveofactant should be mentioned in the abstract. The display of numbers will amplify the effect on reader about overall results of study.

Introduction

1. It is very long. RDS is well studied entity so the history and type of surfactants can be ignored.
2. The utility of functionality scores should be discussed in modern medicine

Methods

1. “A surfactant would be most functional when it required less redosing;”- I would rather use word efficacious than functional in this sentence.
2. Medical referral rate: where are babies referred after surfactant administration.?
3. Sample size calculation for study?
4. How were MABAC and CRITIC computed? Any software?
5. MABAC and CRITIC background calculation and description are not need?

Results

1. Gestational age and Birth weight should be mentioned as mean (standard deviation)
2. The proportion of babies receiving surfactants (all types) were more in babies > 1500 g.
3. The average length of stay was quite short considering the GA and weight. Explanation?
4. DALY/YLL/YLD should be expressed in years.
5. Can the course of babies included in studies be compared? Number of babies needing invasive/non invasive ventilation, shock, IVH, PVL, etc. These all factors can also have bearing on the indicators.

Discussion

1. The evidence on efficacy of surfactants is already there so the studies which have looked at surfactant in a similar perspective (economic efficacy).
2. I am not sure how the cost can be compared from different countries at different time points. Cost is variable and dependent on plethora of factors.
3. The need for mechanical ventilation can be due to multiple reasons or other single etiologic factors than RDS.
